# Fibroblast activation protein is dispensable in the anti-influenza immune response in mice

**DOI:** 10.1371/journal.pone.0171194

**Published:** 2017-02-03

**Authors:** Sioh-Yang Tan, Sumaiya Chowdhury, Natasa Polak, Mark D. Gorrell, Wolfgang Weninger

**Affiliations:** 1 Immune Imaging Program, Centenary Institute for Cancer Medicine and Cell Biology, Newtown, New South Wales, Australia; 2 Sydney Medical School, The University of Sydney, New South Wales, Australia; 3 Molecular Hepatology Laboratory, Centenary Institute for Cancer Medicine and Cell Biology, Newtown, New South Wales, Australia; 4 Department of Dermatology, Royal Prince Alfred Hospital, Camperdown, New South Wales, Australia; Imperial College London, UNITED KINGDOM

## Abstract

Fibroblast activation protein alpha (FAP) is a unique dual peptidase of the S9B serine protease family, being capable of both dipeptidyl peptidase and endopeptidase activities. FAP is expressed at low level in healthy adult organs including the pancreas, cervix, uterus, submaxillary gland and the skin, and highly upregulated in embryogenesis, chronic inflammation and tissue remodelling. It is also expressed by cancer-associated stromal fibroblasts in more than 90% of epithelial tumours. FAP has enzymatic and non-enzymatic functions in the growth, immunosuppression, invasion and cell signalling of tumour cells. FAP deficient mice are fertile and viable with no gross abnormality, but little data exist on the role of FAP in the immune system. FAP is upregulated in association with microbial stimulation and chronic inflammation, but its function in infection remains unknown. We showed that major populations of immune cells including CD4^+^ and CD8^+^ T cells, B cells, dendritic cells and neutrophils are generated and maintained normally in FAP knockout mice. Upon intranasal challenge with influenza virus, FAP mRNA was increased in the lungs and lung-draining lymph nodes. Nonetheless, FAP deficient mice showed similar pathologic kinetics to wildtype controls, and were capable of supporting normal anti-influenza T and B cell responses. There was no evidence of compensatory upregulation of other DPP4 family members in influenza-infected FAP-deficient mice. FAP appears to be dispensable in anti-influenza adaptive immunity.

## Introduction

Fibroblast activation protein alpha (FAP), previously known as seprase, is a member of the S9B serine protease family, which comprises dipeptidyl peptidases uniquely capable of cleaving a post-proline peptide bond [1, 2]. The members of this family include DPP4/CD26, DPP8 and DPP9. FAP has closest homology to DPP4, with which it shares 51% identity in amino acid sequence in the mouse, but FAP is unique in the DPP4 family as it is also an endopeptidase[3]. Both the dipeptidase and endopeptidase activities of FAP depend on the catalytic triad of serine (S624), aspartate (D702) and histidine (H734) in its extracellular domain [4, 5]. Dimerisation and glycosylation are required for FAP to be proteolytically active [6, 7]. FAP has also been found to hetero-oligomerise with DPP4 to form complexes required for lung fibroblast migration in collagen [8].

Both tumour-promoting and tumour suppressing roles of FAP by enzymatic and non-enzymatic means have been described [5, 9–15]. FAP is present in lipid rafts and invadopodia alluding to a role in cell migration and invasion [16]. The endopeptidase activity of FAP allows it to cleave α2-antiplasmin, collagen and gelatin [17–19], and to cleave and inactivate the human stress-induced hormone Fibroblast Growth Factor (FGF)-21 [20–22]. Neuropeptide Y (NPY), peptide YY, B-type natriuretic peptide and substance P are natural substrates of FAP by virtue of its dipeptidyl peptidase activity [23].

FAP is expressed on the cell surface and as a soluble form [4, 18]. *In silico* expression analysis and subsequently bioluminescence imaging and zymogenic assays found widespread low-level basal FAP expression in various organs in the mouse, baboon and human [24–26]. Importantly, a large body of research in cancer and inflammation concur that FAP expression defines reactive stromal fibroblasts at sites of inflammation and remodelling [14, 27, 28]. FAP expression is upregulated in sites of chronic inflammation [14, 28, 29] such as osteoarthritis [30], rheumatoid arthritis [31, 32], Crohn’s disease [33], chronic liver cirrhosis [34], subtypes of atheromata [35], idiopathic pulmonary fibrosis [36] and keloids [37]. FAP is also increased in remodelling tissue for example in readsorbing tadpole tail [38] and healing wounds [27]. Remarkably, FAP is highly expressed on cancer-associated fibroblasts in more than 90% of epithelial tumours [1, 24, 27]. FAP^+^ stromal cells in distinct organs share similar transcriptomic profiles suggesting a common mesenchymal origin [26]. In addition, FAP is expressed in some tumour cells [39] and in a minor population of intratumoural CD45^+^F4/80^hi^CCR2^+^CD206^+^ M2 macrophages [40]. FAP^+^ cells in different microenvironments have different secretome profiles and distinct roles. In the tumour microenvironment, depletion of FAP^+^ cells enhances intratumour T cell infiltration [41], relieves metabolic stress on and delays the exhaustion of CD8^+^ T cells [42] and enhances TNF and IFN-γ dependent hypoxic killing of tumour cells by T cells [43]. FAP^+^ stromal cells may exert their immunosuppressive function by production of CXCL12 [41], inactivation of CCL2 [44] or recruitment of myeloid derived suppressive cells (MDSC) via the CCL2-STAT3 signalling axis [45]. Furthermore, FAP^+^ stromal cells maintain the deposition of extracellular matrix and glycosaminoglycan and promote angiogenesis in the tumour microenvironment such that their ablation improves intratumoural uptake of chemotherapeutic drugs and suppresses tumour growth [9, 46, 47]. On the other hand, immunosuppression by FAP^+^ tumour associated macrophages is mediated by heme-oxygenase-1 (HO-1) [40].

As a genetically stable molecule expressed on the cell surface and selectively upregulated on reactive stromal fibroblasts in a broad range of cancers, FAP is an attractive target for antitumour therapy [9, 47–49]. Nonetheless, the biology of FAP itself remains inadequately understood. A FAP knockout mouse strain has been made by deleting exon 4, intron 4, and part of exon 5 [50, 51]. This FAP knockout mouse develops and reproduces normally and shows no increase in the incidence of cancer [51], suggesting that FAP is not essential during development [52]. Detection of low-level FAP in the bone marrow suggests that it may affect the hematopoietic system and ontogeny of immune cells [26, 43], but the immunological phenotype of FAP knockout mice has not been described in detail. Furthermore, the role of FAP in infection remains largely uncharacterised. In chronic infection with hepatitis C virus (HCV), FAP expression occurs at necroinflammatory sites and its intensity correlates with severity of hepatic fibrosis [34]. In uterine fibroid-derived fibroblasts and myometrial fibroblasts, LPS induces FAP protein expression in a TLR-4/NF_k_B dependent pathway [50]. These studies suggest that FAP activity is modulated in infection where it may have an immunopathologic role. It is conceivable that anti-FAP treatment may alter immune defence in cancer patients, a cohort that is highly susceptible to untoward consequences of infection.

To further investigate the role of FAP in an infectious setting, we compared immune parameters in FAP knockout and wildtype mice during influenza virus infection. We found that FAP expression was upregulated in the lungs and lung-draining mediastinal lymph nodes. Despite this increase due to infection, there was no evidence of alteration in morbidity and mortality, antigen-specific T cell proliferation and cytokine production, nor the anti-influenza antibody response in FAP knockout mice. These findings indicate that FAP activity is not required for the anti-influenza adaptive immune response.

## Materials and methods

### Mice

FAP knockout mice [51] on a C57BL/6J background were a gift from Boehringer Ingelheim Pharma Kg, Germany and were backcrossed onto C57BL/6J for more than seven generations. C57BL/6J wildtype (WT) mice were purchased from Animal Resources Centre, Perth, WA, Australia. tdTomato x OT-I TCR transgenic mice were generated by crossing tdTomato mice on a C57BL/6 background with OT-I TCR transgenic mice [52] for more than eight generations. All mice were maintained in the animal facilities of Centenary Institute in compliance with appropriate regulations. Age- and sex-matched FAP knockout and C57BL/6J wildtype (WT) were co-housed in all experiments. Experiments were carried out with approval of the Animal Ethics Committee, University of Sydney and the Royal Prince Alfred Hospital.

### Antibodies and reagents

The following monoclonal antibodies conjugated to biotin or various fluorochromes are used in this study: B7-H1/PD-L1/CD274 (1-111A), CD45 (30-F11), CD86 (GL1), CD103 (2E7) (eBioscience, San Diego, CA, USA), B220 (RA3-6B2), CD3 (145-2C11), CD4 (GK1.5), CD8 (53–6.7), CD11b (M1/70), CD11c (HL3), CD16/CD32 (24G2), CD25 (PC61), CD44 (IM7), CD45.1 (A20), CD45.2 (104), CD62L (MEL-14), Gr-1 (RB6-8C5), IL-2 (JES6-5H4), IFN-γ (XMG1.2), rat IgG2ak, rat IgG2bk, I-A/I-E (M5114), NK1.1 (PK136), siglec-F (E50-2440) (BD Biosciences, San Diego, CA, USA). Dead cell exclusion was done by 4’,6-diamidino-2-phenylindole (DAPI) or live/dead Aqua staining (Invitrogen/ Thermo Fisher Scientific, Waltham, MA USA).

### Flow cytometry

Single cell suspensions for flow cytometry were prepared from spleens, peripheral lymph nodes (pLN), mesenteric LN (mLN), mediastinal LN (mdLN), thymi or lungs as previously described [53]. Cells were stained and analysed on a FACS LSR II or Fortessa cytometer (BD Biosciences). Flow data was analysed using FlowJo softwares (Tree Star, Ashland, OR, USA).

### Intracellular cytokine staining

Single cell suspensions from the organs of interest were plated at 2x10^6^ cells per well in 96 well U bottom tissue culture plate in T cell medium (RPMI 1640; (Gibco), 10% FCS, 1 mM sodium pyruvate, 10 mM HEPES, 100 U/ml penicillin, 100 mg/ml streptomycin and 50 mM 2-mercaptoethanol) containing 20 U/mL recombinant murine interleukin-2 (R&D Systems, Minneapolis, MN, USA) and 0.66 μl/mL GolgiStop (BD Biosciences) with 1 μg/mL SIINFEKL peptide (Auspep Pty Ltd, Tullamarine, VIC, Australia). Cells were incubated for 6 hours at 37°C. At the end of the incubation, cells were surface-stained with fluorescently conjugated primary antibodies then fixed and permeabilised using the CytoFix/ CytoPerm Kit (BD Biosciences) and stained with anti-cytokine antibodies. Cells were run on a FACS LSR II or Fortessa cytometer (BD Biosciences). Flow data was analysed using FlowJo softwares (Tree Star, Ashland, OR, USA). Gating strategy to identify cytokine-producing cells is shown in **S1 File**.

### Intranasal infection with influenza virus

Influenza A/PR/8 and A/PR/8-OVA, the latter expressing ovalbumin peptide 257–264, were kind gifts of Stephen Turner, University of Melbourne, Australia [54]. Female mice between 8 to 12 weeks of age were used for experiments. Prior to infection, mice were anesthetised with ketamine (80 mg/kg) and xylazine (10 mg/kg). Each mouse received a 25 μL intranasal inoculation of PR/8 virus or PR/8-OVA in PBS as described previously [52, 55]. To assess anti-influenza T cell responses, single cell suspensions were prepared from pooled lymph node cells of OT-I TCR or tdTomato x OT-I TCR transgenic donor mice and 1 to 2x10^6^ cells were intravenously injected into FAP knockout or WT recipients one day prior to intranasal influenza infection. On day 7 post-infection, cells were harvested and stained for flow cytometric analysis as described.

### Real time quantitative PCR (qPCR)

Real time qPCR by Taqman gene expression assays was performed using the Stratagene Mx3000P™ System (Stratagene, La Jolla, CA, USA) as described previously [56]. Briefly, total RNA was extracted from tissue using TRIzol reagent (Invitrogen) and 0.5 μg of the extracted RNA was reverse transcribed to cDNA using Superscript VILO cDNA synthesis kit (Invitrogen). The gene expression levels were analysed in duplicate using mouse FAP Taqman assay reagent (Assay identification number Mm01329176_m1), mouse DPP4 (Mm00494548_m1), DPP8 (Mm00547049_m1) and DPP9 (Mm00841122_m1) (Applied Biosystems, Forster City, CA, USA) and a standard curve of serially diluted known numbers of molecules of the gene and then normalised relative to mouse hypoxanthine guanine phosphoribosyl transferase (HPRT) (Mm01545399_m1) or 18S (Hs99999901_s1) (Applied Biosystems, CA, USA). For quantitation of influenza viral RNA, lung RNA was isolated using the PureLink RNA Mini kit (Ambion/ Thermo Fisher Scientific). Total RNA was quantified using the Qubit RNA BR Assay Kit (Molecular Probes/ Life Technologies, Carlsbad, CA, USA) and a Qubit 3.0 Fluorimeter (Life Technologies). The cDNA template was prepared using Superscript as above. Influenza A RNA was quantified in triplicate using a TaqMan Influenza A Assay primer and probe set (cat. no. 4441242C) and the QuantStudio^TM^ 12K Flex Real-Time PCR System (Applied Biosystems), and Ct analysis using ExpressionSuite v1.0 (Applied Biosystems).

### Measurement of anti-influenza antibodies in the serum

Anti-haemagglutinin (HA) antibody titres in the sera were determined using the R&D indirect ELISA system (R&D Systems, Minneapolis, MN) with modification. Briefly, 96 well U-bottom vinyl non-treated Serocluster microplates (Costar, Corning NY) were coated with 100 ng Influenza A H1N1 (A/PR/8/1934) HA1 protein (Sino Biological, PR China) per well overnight at room temperature. All subsequent incubations were carried out at room temperature and plates were washed thrice with complete removal of residual fluid between incubation steps. Two-fold serially diluted sera were added in duplicates and incubated for 2 hours. After washing, horseradish peroxidase-conjugated goat anti-mouse IgG (Santa Cruz, CA) was added at 1:12000 for 2 hours. TMB substrate (Sigma-Aldrich Australia) was added and optical density read off a PolarStar microplate reader (BMG Labtech, Germany). The antibody titre was determined as the reciprocal of the highest dilution of the sample that yielded a reading of 2 SD above the mean of uninfected sera. To determine the level of anti-influenza neutralising antibodies, heat-inactivated and adsorbed sera from uninfected control and infected mice were assayed for haemagglutination inhibition (HI) as described [57].

### Statistical analysis

The Student’s t-test was used to analyse the difference in weight loss between FAP knockout and C57BL/6J wildtype mice in response to influenza infection. For all other experiments comparing two groups, the Mann-Whitney non-parametric test was used. The Kruskal-Wallis H-test was applied to test for statistical significance where there were more than two groups. Significance was considered if p < 0.05.

## Results

### Normal development and maintenance of major populations of immune cells in FAP knockout mice

**Fig 1:**
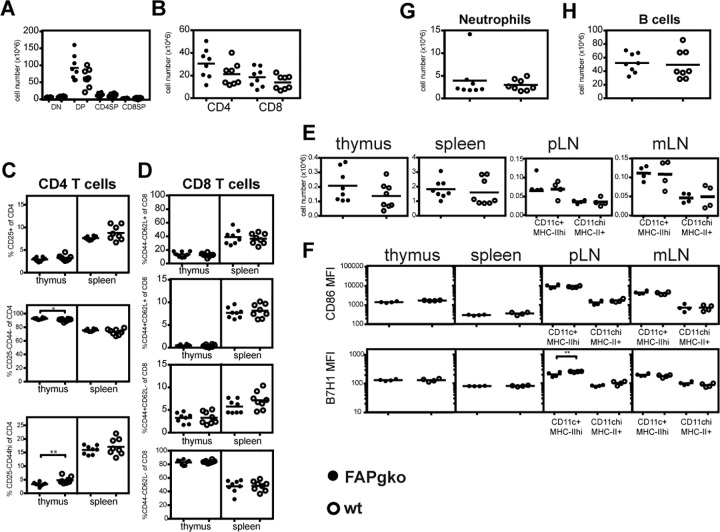
Effects of genetic deficiency of FAP on the numbers and phenotype of leukocytes at steady state. Organs were harvested from FAP knockout (gko; closed symbols) and wildtype (WT; open symbols) C57BL/6 mice. **A.** The numbers of thymic CD4^-^CD8^-^ double negative (DN), CD4^+^CD8^+^ double positive (DP) CD4^+^CD8^-^ and CD8^+^CD4^-^ single positive (SP) populations. **B.** The numbers of splenic CD4^+^ and CD8^+^ T cells. **C.** The percentages of CD44^-^ naïve, CD25^+^ regulatory and CD44^hi^ activated/ memory CD4^+^ T cells in the thymus and the spleen. **D.** The percentages of CD8^+^ T cell subsets gated according to their expression of CD44 and CD62L in the thymus and the spleen. **E.** The numbers of dendritic cells (DC) in the spleen, peripheral LN (pLN), mesenteric LN (mLN) and thymus in FAP knockout and WT mice at steady state. **F.** The geometric mean fluorescence index (MFI) of surface expression of CD86 (top) and B7H1/PD-L1 (bottom) on DC in the spleen, pLN, mLN and thymus. **G.** The numbers of splenic CD11b^+^Ly6G^+^ neutrophils. **H.** The numbers of splenic CD19^+^ B cells. Each symbol represents one mouse and the bar represents the mean. Except for part **F**, results were pooled from two independent experiments of n = 4 mice each. For **F**, results from one of three experiments (n = 4 mice each) are shown. Statistical significance was tested using the Mann–Whitney test. * p<0.05, ** p<0.01.

To determine if genetic deficiency of FAP affected the development and maintenance of T cells, dendritic cells (DC), granulocytes and B cells, we compared the numbers and/or phenotype of these cells in the thymus, spleen and lymph nodes in FAP knockout and wildtype (WT) C57BL/6J mice using flow cytometry. In the thymus of FAP knockout mice, the numbers of all subsets including CD4^-^CD8^-^ double negative, CD4^+^CD8^+^ double positive, CD4^+^ and CD8^+^ single positive thymocytes were unchanged (**Fig 1A** & **S2 File Part A**). There were also no significant differences between the proportion of thymic DN subsets, delineated by surface expression of CD25 and CD44 (data not shown). In the spleen, FAP knockout and wildtype mice had similar numbers of CD4^+^ and CD8^+^ T cells (**Fig 1B** & **S2 File Part B**). Using cell surface expression of the activation markers CD25 and CD44 to segregate CD4^+^ T cells into CD25^+^CD44^int^ regulatory, CD25^-^CD44^-^ naive or CD25^-^CD44^hi^ activated subsets, we observed a modest, yet significant increase of activated cells in the thymus, concomitant with a decrease in the naive subset (**Fig 1C**). Nonetheless, the distribution of these CD4^+^ subsets did not differ in the spleen between the two strains of mice. Consistently, FAP deficiency did not alter the activation profiles of CD8^+^ T cells in both the thymus and the spleen, based on analysis of their surface expression of CD44 and CD62L (**Fig 1D**). These data indicate that genetic deficit of FAP does not perturb the generation of T cells in the mouse thymus nor their maintenance in the periphery.

The total number of DC in the thymus and spleen of FAP knockout mice was not significantly different from wildtype animals, nor were the numbers of CD11c^+^MHC-II^high^ migratory DC and CD11c^high^MHC-II^+^ resident DC in the lymph nodes (**Fig 1E**). Beside a small reduction in the expression level of B7-H1/PD-L1 in the CD11c^+^ MHC II^high^ migratory DC subset of peripheral lymph nodes, there was no difference between FAP knockout and wildtype mice in expression of costimulatory molecules CD86 and B7-H1 (**Fig 1F**). FAP knockout and wildtype mice also did not differ in the splenic numbers of neutrophils (**Fig 1G**) and B cells (**Fig 1H**). Based on the analysis of the numbers and phenotype of leukocytes in the steady-state, we concluded that FAP deficiency did not lead to any overt abnormality in the development and maintenance of the major immune cell subsets.

### FAP gene deficiency does not affect anti-influenza T and B cell responses

**Fig 2:**
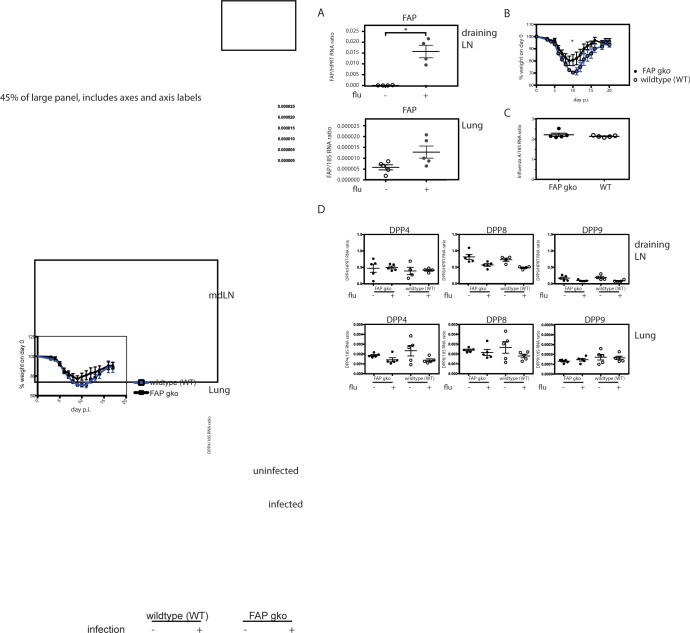
The pathologic response of FAP knockout (gko) versus wildtype (WT) mice to intranasal influenza infection and the associated changes in expression of DPP4 family members. FAP knockout (gko; closed symbols) and WT (open symbols) mice were intranasally infected with 50 pfu PR/8 influenza virus. **A.** Real-time quantitative PCR results showing relative expression of FAP normalised to the housekeeping gene *hprt* in the mediastinal lymph nodes (mdLN), and normalised to the *18S RNA* gene in the lungs (bottom) of uninfected (-) and infected (+) WT mice on day 7 post-infection. Statistical significance was tested using Mann-Whitney test. **B.** Weight loss response in influenza-infected 10-week old female FAP knockout (closed symbols) and WT (open symbols) mice (n = 7). Results from one of three replicate experiments are shown. Statistical significance was tested using Student’s t-test. * p< 0.05. **C.** Real-time quantitative PCR results showing relative expression of influenza virus (Inf A), normalised to the 18S RNA gene in the lungs of infected FAP knockout (closed symbols) and WT (open symbols) mice on day 7 post-infection (n = 5 per group). Inf A expression was not detected in uninfected lungs. Each symbol represents data from one mouse and the bar represents the mean±SEM. Statistical significance was tested using the Mann-Whitney test. **D.** Expression of DPP4 family members DPP4, DPP8 and DPP9 in uninfected and infected FAP knockout (closed symbols) and WT (open symbols) mice. Each symbol represents data from one mouse and the bar represents the mean±SEM. Results from one of two experiments are shown. Statistical significance was tested using the Mann-Whitney test.

Intranasal infection with a sublethal dose of influenza virus H1N1/PR/8 induced marked upregulation of FAP expression in the lungs and lung-draining mediastinal lymph nodes of wildtype mice (**Fig 2A**). However there was no difference in the kinetics of infection-induced weight loss in FAP knockout and wildtype mice, except on day 10 post-infection, when wildtype showed slightly lower body weight relative to FAP knockout mice (**Fig 2B** & **S1 Table**). Furthermore, weight loss of the two groups also did not differ in additional independent experiments using different infectious doses (**S3 File**). The viral load in the lungs of FAP knockout mice did not differ from wildtype, as assessed by viral RNA level on day 7 post-infection (**Fig 2C**). Together, these observations indicate that the absence of FAP did not impair the anti-influenza response.

To investigate whether other family members of the DPP4 family compensated for the absence of FAP, we measured their expression using real-time PCR. Neither DPP4, DPP8 nor DPP9 were significantly upregulated, arguing against a compensatory effect (**Fig 2D**).

**Fig 3:**
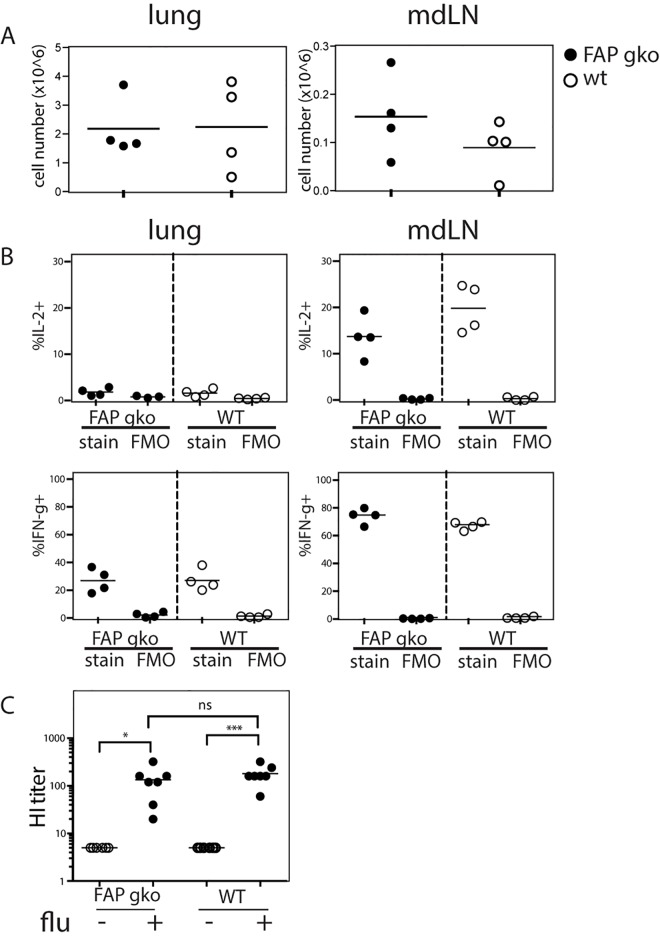
T and B cell responses to influenza in FAP knockout (gko) and wildtype (WT) mice. **A & B.** FAP knockout (gko; closed symbols) and wildtype (WT; open symbols) mice received intravenous transfer of donor OT-1 cells and on the next day were intranasally inoculated with 100 pfu PR/8-OVA influenza virus. Organs were obtained on day 7 post-infection. **A.** The numbers of donor cells recovered from recipient lungs and mediastinal lymph nodes (mdLN) on day 7 post-infection. **B.** Cells harvested from the lungs and mdLN were restimulated *in vitro* in the presence of SIINFEKL peptide and the production of IL-2 (top) and IFN-γ (bottom) by donor OT-I cells was quantified by flow cytometry. The percentage of positive events in stained samples and fluorescence minus one (FMO) negative controls are shown. Each data point represents an individual mouse and the bar represents the mean. Results from one of three experiments are shown. Statistical significance was tested using the Mann–Whitney test. **C.** FAP knockout and WT mice were intranasally infected with 50 pfu PR/8 virus and sera were harvested on day 21 post-infection. Neutralising anti-influenza antibody titres in the sera were measured using haemagglutination inhibition (HI) assay. Each data point represents an individual mouse and the bar represents the mean. Statistical significance was tested using Kruskal-Wallis test with Dunn’s multiple comparison test. * p<0.05, *** p<0.001, ns not significant.

CD8^+^ T cells are required for viral clearance during influenza virus infection [58, 59]. We used an adoptive transfer model to characterise the effect of FAP deficiency on antigen-specific anti-influenza T cell responses. To this end, we transferred OT-I TCR transgenic cells into FAP knockout or wildtype recipients, which were infected with a PR/8 influenza strain engineered to carry the ovalbumin (OVA)257-264 antigen, cognate antigen for the OT-I TCR, in the neuraminidase stalk [54]. As lymphocytes have negligible FAP enzymatic activity [5] the use of T cells from FAP^+^ donors should not reverse the FAP deficit in FAP knockout recipients. On day 7 post-infection, donor OT-I cells proliferated, and differentiated into CD44^+^CD62L^-^ effector T cells to a similar extent in FAP knockout and WT mice (**Fig 3A**). PR/8-OVA infection also did not induce differential changes in host T cell number of the two groups (**S4 File**). In addition, comparable production of the cytokines IL-2 and IFN-γ were observed in both groups of mice (**Fig 3B**). These results indicate that the proliferation and differentiation of antigen-specific CD8^+^ T cells in influenza infection were independent of FAP expression by the host. Finally, the role of FAP in anti-influenza antibody production was examined in mice infected with 50 pfu (**Fig 3C**) and 25 pfu **(S5 File**) influenza PR/8 virus. Antibody production was not affected by the absence of FAP. We conclude that deficiency of FAP does not compromise the T and B cell responses in acute influenza infection *in vivo*.

## Discussion

FAP is a dipeptidyl peptidase and endopeptidase that has closest homology with DPP4/CD26. Both FAP knockout and DPP4 knockout mice have no gross abnormality when untreated [51, 60]. While DPP4 knockout mice have altered proportions of CD4 T cells, NK cells and NKT cells, altered cytokine and antibody production [61] and heightened response to allergenic sensitisation [62], we found no difference between FAP knockout and wildtype mice in CD4^+^ and CD8^+^ T cells, B cells, dendritic cells and neutrophils. T cells and dendritic cells in FAP knockout mice were phenotypically indistinguishable from their wildtype counterparts. While DPP4 is known to be a costimulator of T cell activation and cleaves multiple chemokines FAP poorly cleaves known chemokine targets of DPP4 and its immunological role is largely unknown [23, 63]. Recently, Denton *et al* described a population of FAP^+^gp38^+^CD31^-^ stromal cells in the lymph nodes that regulate naive T cell immigration through production of the chemokines CCL19 and CCL21 [64]. The ablation of such cells prior to infection diminishes the number of naive T cells in the nodes and compromises the T and B cell responses [64]. However, unaltered numbers of T cells in the lymph nodes of FAP knockout mice as seen in our studies indicates that FAP itself is unlikely to be directly required for this role (**Fig 1**). These observations indicate that FAP deficiency does not impair the development and maintenance of immune cells. The disparity between FAP expression and its requirement in physiological processes has been reported in other settings [43].

FAP plays a role in lung tissue homeostasis and FAP deficiency exacerbates fibrosis in lung injury models [65]. However, in our model of intranasal influenza infection, FAP deficiency did not affect viral clearance in the lung and did not worsen the immunopathological response to infection as assessed by the combined parameters of weight loss, T cell and B cell functions. The lack of impairment in anti-influenza immunity is not due to a compensatory increase of the level of another protease in the DPP4 family. Our results suggest that FAP activity is not required for anti-influenza adaptive immunity. While our data supports the notion that ablation of FAP activity does not adversely affect the anti-influenza response, it does not contradict earlier reports of a requirement for FAP^+^ cells in regulating the number of naive T cells in the lymph node via chemokine production [64].

While there is strong interest to develop FAP targeting anti-cancer strategies for the clinic [43, 46–49, 66–68], it is of concern that studies looking at off-tumour effects of FAP-targeting regimens have yielded mixed results [26, 46, 47, 69–72]. Cancer patients are highly susceptible to the long-term complications of influenza infection [73, 74]. As such, understanding the role of FAP in infection is warranted to inform FAP targeting strategies in these patients. We showed that although the expression of FAP is increased in the lungs and lung-draining lymph nodes in influenza infection, its absence did not alter the antiviral CD8^+^ T cell and B cell responses, nor affected the course of recovery in infected mice. Secondary sublethal infection with a heterologous strain of influenza also did not exacerbate weight loss response in FAP knockout mice when compared to wildtype (data not shown).

Recent studies collectively demonstrated that given a FAP-targeting moiety of the right specificity, in combination with targeting of tumour antigen [75] or immune checkpoints [42], and optimal modes of delivery, FAP targeting may potentially result in clinically beneficial tumour-specific outcome with minimal off-tumour on-target toxicity. Better understanding of the biology of FAP and FAP-expressing cells is needed to improve the design of FAP targeting regimens for cancer treatment. Knowledge of the enzymatic and non-enzymatic physiological roles of FAP, and identification of its natural substrates, will be instrumental to better harness the usability of this protein in the clinic [66].

## Supporting information

S1 File Gating strategy to identify cytokine-producing donor CD8^+^ T cells in the lung. Dead cells were excluded by Aqua dye, followed by exclusion of lung non- T cells with high side scatter. CD8^+^CD4^-^ cells were gated, and donor cells discriminated from host cells by tdTomato positivity. Fluorescence minus one (FMO) samples were used to set the gate for cytokine-producing IFN-γ^+^ and IL-2^+^ CD8^+^ donor T cells.
(PDF)

S2 File Numbers and percentages of T cell populations in FAP knockout and C57BL/6 wildtype (WT) mice. **A.** Figure shows the number (left) and percentages (right) of thymic CD4^-^CD8^-^ double negative (DN), CD4^+^CD8^+^ double positive (DP) CD4^+^CD8^-^ and CD8^+^CD4^-^ single positive (SP) populations in FAP knockout (closed symbols) and C57BL/6 wildtype (WT; open symbols) mice. **B.** The numbers (left) and percentages (right) of splenic CD4^+^ and CD8^+^ T cells. Each symbol represents one mouse and the bar represents the mean. Results were pooled from two independent experiments of n = 4 mice each. Statistical significance was tested using the Mann-Whitney U test. * p<0.05. Note that the left panels of this figure are from **Fig 1**.
(PDF)

S3 File Kinetics of weight loss post-influenza infection in FAP knockout and C57BL/6 wildtype (WT) mice. 5 mice per group were infected with 50 pfu (**A**) or 25 pfu (**B**) influenza PR/8. Graphs show the mean (± SEM) percentage body weight post- infection in proportion to day 0. Results were statistically tested with Student’s t- test. There was no statistically significant difference between infected FAP knockout (closed symbols) and WT (open symbols) mice in both **A** and **B**.
(PDF)

S4 File Numbers of host CD4^+^ and CD8^+^ T cells in infected mice. The numbers of host CD4^+^ and CD8^+^ T cells in the lungs (left) and mediastinal lung-draining lymph nodes (right) of infected FAP knockout (closed symbols) and wildtype (WT; open symbols) mice are shown. Mice received adoptive transfer of OT-I T cells on day -1 and were intranasally infected with 100pfu PR/8-OVA influenza virus on day 0. On day 7 mice were euthanised and tissues harvested for flow cytometry. Each dot represents one mouse and the bar represents the mean. Data were subjected to Mann-Whitney statistical test. There was no statistically significant difference between FAP knockout and WT mice in the number of host CD4^+^ and CD8^+^ T cells in both the lungs and mediastinal lymph nodes.
(PDF)

S5 File Anti-influenza antibody response in FAP knockout and WT mice. FAP knockout (closed symbols) and WT (open symbols) mice were intranasally infected with 25 pfu influenza PR/8 virus and sera were harvested on day 12 post-infection. Each data point represents an individual mouse and the bar represents the mean. Statistical significance was tested using the Mann-Whitney test. *** p<0.001. **A**. Anti-haemagglutinin (HA) antibody titres in mouse sera were measured using indirect ELISA. **B**. Neutralising anti-influenza antibody titres in the sera were measured using haemagglutination inhibition assay.
(PDF)

S1 Table Mean percentage body weight of infected FAP knockout and wildtype mice. Female 10-week old FAP knockout mice and C57BL/6 wildtype (WT) mice were intranasally infected with 50 pfu influenza PR/8 virus (n = 7). Table shows the mean percentage body weight ± SEM of infected mice in proportion to body weight on day 0, from day 7 to 16 post-infection. The data was statistically analysed with Student’s t- test. ns non-significant; * p<0.05
(PDF)

## Abbreviations

B7-H1: B7 homolog 1
BM: bone marrow
CD: Cluster of Differentiation
DC: dendritic cells
DAPI, 4’: 6-diamidino-2-phenylindole
DPP: dipeptidyl peptidase
FAP: fibroblast activation protein alpha
HCV: hepatitis C virus
IFN: interferon
LN: lymph node
mdLN: mediastinal lymph node
pLN: peripheral lymph node
MHC: major histocompatibility complex
OVA: ovalbumin
PD-L1: Programmed cell death 1 ligand 1
TCR: T cell receptor
TNF: tumour necrosis factor
