# Supplementary figures and images for: Fibroblast activation protein is dispensable in the anti-influenza immune response in mice
Source: PLoS One. 2017 Feb 3;12(2):e0171194. doi: 10.1371/journal.pone.0171194 (PMC5291439; doi:10.1371/journal.pone.0171194)

S1 File. Gating strategy to identify cytokine-producing donor CD8+ T cells in the lung

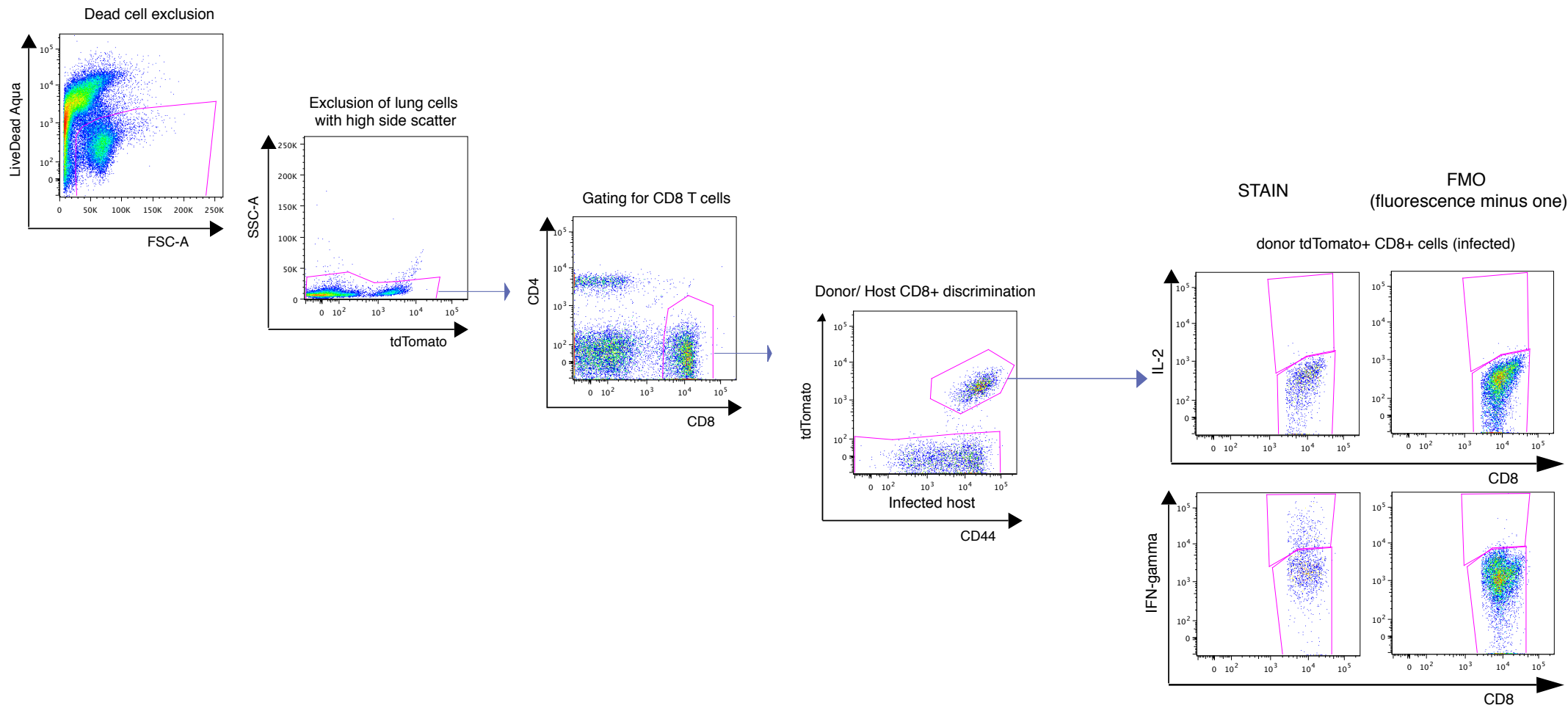

Supplement: S1 File — Dead cells were excluded by Aqua dye, followed by exclusion of lung non- T cells with high side scatter. CD8+CD4- cells were gated, and donor cells discriminated from host cells by tdTomato positivity. Fluorescence minus one (FMO) samples were used to set the gate for cytokine-producing IFN-γ+ and IL-2+ CD8+ donor T cells. (PDF) [file pone.0171194.s001.pdf]

## S2 File

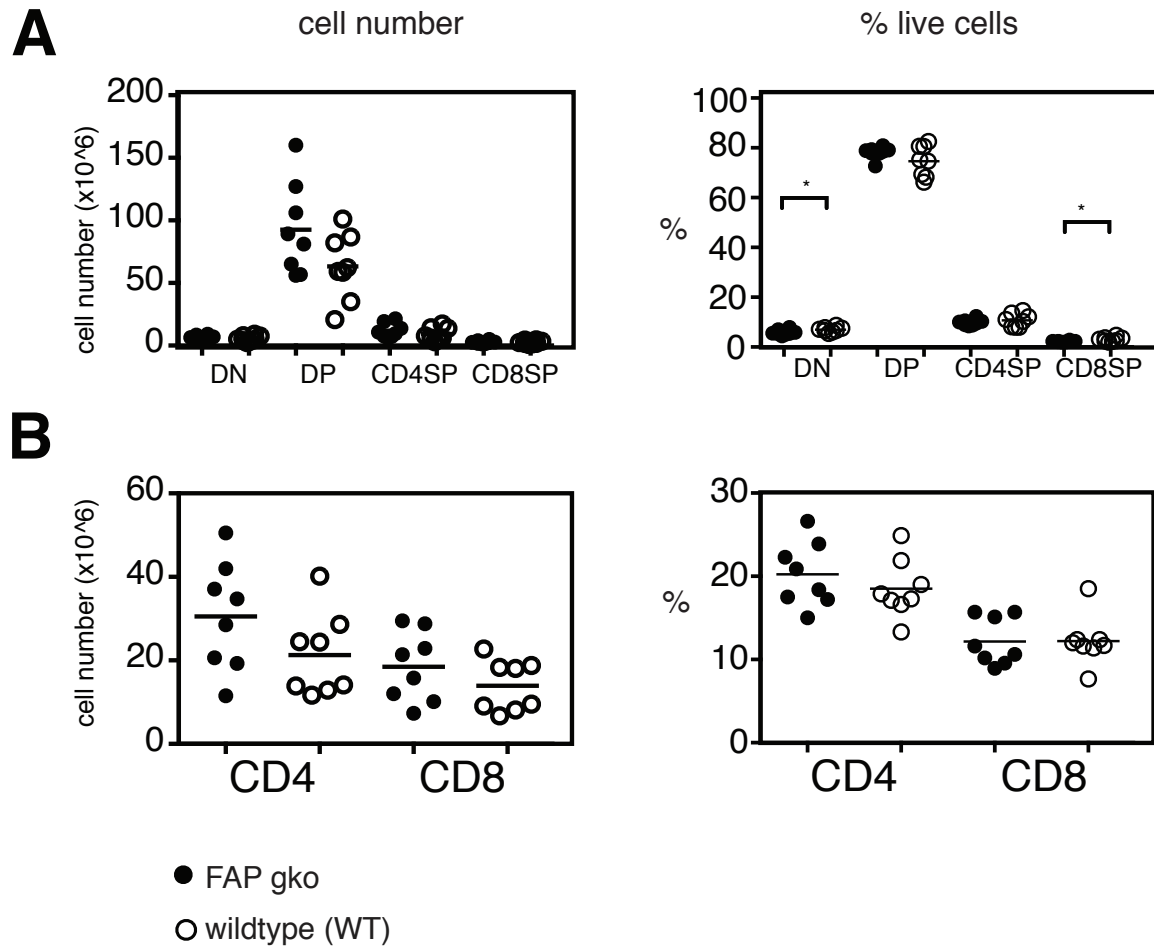

Supplement: S2 File — A. Figure shows the number (left) and percentages (right) of thymic CD4-CD8- double negative (DN), CD4+CD8+ double positive (DP) CD4+CD8- and CD8+CD4- single positive (SP) populations in FAP knockout (closed symbols) and C57BL/6 wildtype (WT; open symbols) mice. B. The numbers (left) and percentages (right) of splenic CD4+ and CD8+ T cells. Each symbol represents one mouse and the bar represents the mean. Results were pooled from two independent experiments of n = 4 mice each. Statistical significance was tested using the Mann-Whitney U test. * p<0.05. Note that the left panels of this figure are from Fig 1. (PDF) [file pone.0171194.s002.pdf]

## S3 File

**A**

50 pfu

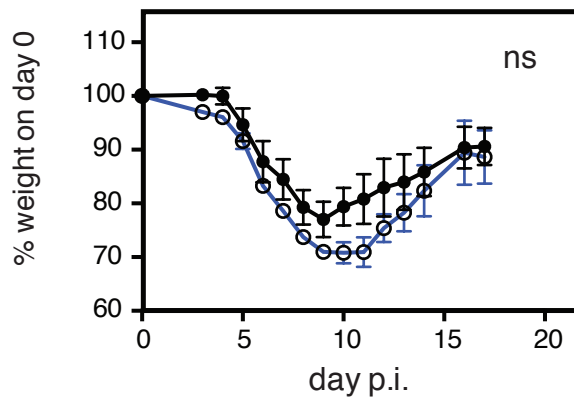

**B**

25 pfu

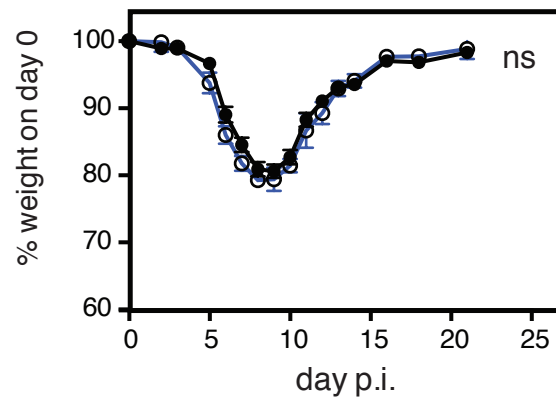

● FAPgko

○ wt

Supplement: S3 File — 5 mice per group were infected with 50 pfu (A) or 25 pfu (B) influenza PR/8. Graphs show the mean (± SEM) percentage body weight post- infection in proportion to day 0. Results were statistically tested with Student’s t- test. There was no statistically significant difference between infected FAP knockout (closed symbols) and WT (open symbols) mice in both A and B. (PDF) [file pone.0171194.s003.pdf]

## S4 File

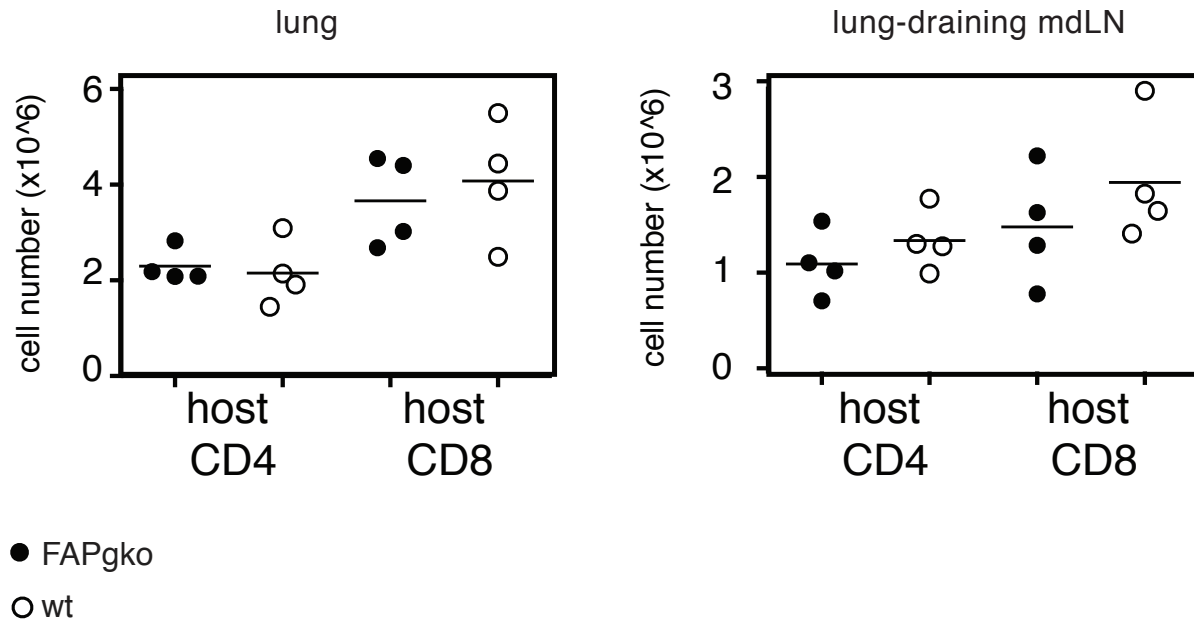

Supplement: S4 File — The numbers of host CD4+ and CD8+ T cells in the lungs (left) and mediastinal lung-draining lymph nodes (right) of infected FAP knockout (closed symbols) and wildtype (WT; open symbols) mice are shown. Mice received adoptive transfer of OT-I T cells on day -1 and were intranasally infected with 100pfu PR/8-OVA influenza virus on day 0. On day 7 mice were euthanised and tissues harvested for flow cytometry. Each dot represents one mouse and the bar represents the mean. Data were subjected to Mann-Whitney statistical test. There was no statistically significant difference between FAP knockout and WT mice in the number of host CD4+ and CD8+ T cells in both the lungs and mediastinal lymph nodes. (PDF) [file pone.0171194.s004.pdf]

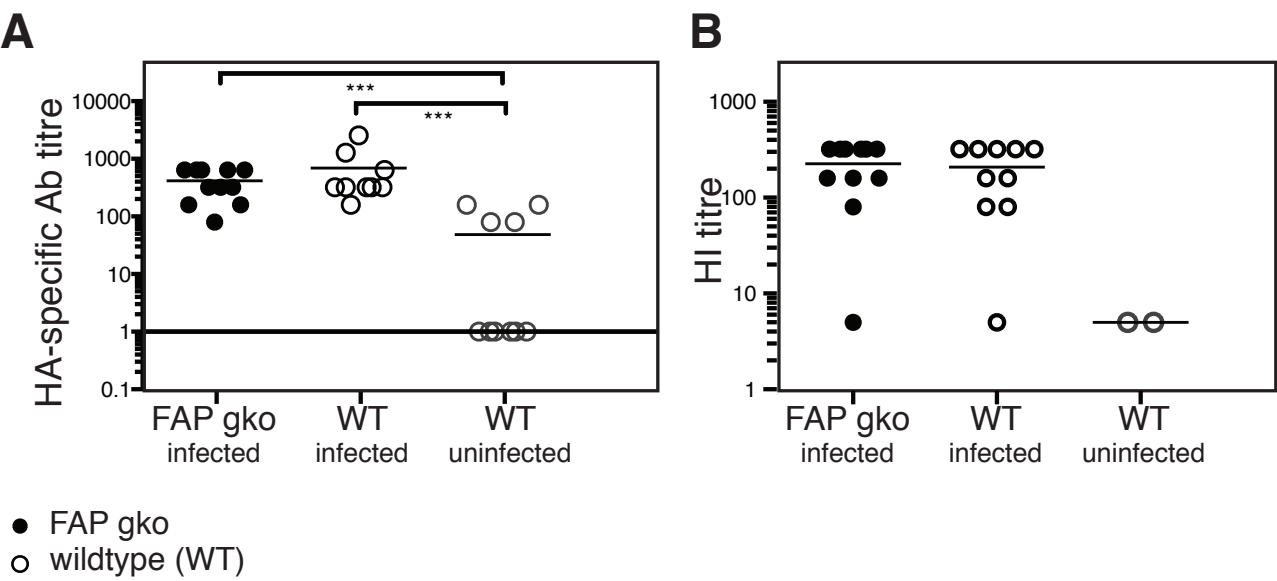

Supplement: S5 File — FAP knockout (closed symbols) and WT (open symbols) mice were intranasally infected with 25 pfu influenza PR/8 virus and sera were harvested on day 12 post-infection. Each data point represents an individual mouse and the bar represents the mean. Statistical significance was tested using the Mann-Whitney test. *** p<0.001. A. Anti-haemagglutinin (HA) antibody titres in mouse sera were measured using indirect ELISA. B. Neutralising anti-influenza antibody titres in the sera were measured using haemagglutination inhibition assay. (PDF) [file pone.0171194.s005.pdf]
