# Supplementary material for: Fibroblast activation protein is dispensable in the anti-influenza immune response in mice
Source: PLoS One. 2017 Feb 3;12(2):e0171194. doi: 10.1371/journal.pone.0171194 (PMC5291439; doi:10.1371/journal.pone.0171194)
Supplement: S1 Table — Female 10-week old FAP knockout mice and C57BL/6 wildtype (WT) mice were intranasally infected with 50 pfu influenza PR/8 virus (n = 7). Table shows the mean percentage body weight ± SEM of infected mice in proportion to body weight on day 0, from day 7 to 16 post-infection. The data was statistically analysed with Student’s t- test. ns non-significant; * p<0.05 (PDF) [file pone.0171194.s006.pdf]

S1 Table. Mean percentage body weight of infected FAP knockout and wildtype mice. Female 10-week old FAP knockout mice and C57BL/6 wildtype (WT) mice were intranasally infected with 50 pfu influenza PR/8 virus (n=7). Table shows the mean percentage body weight ± SEM of infected mice in proportion to body weight on day 0, from day 7 to 16 post-infection. The data was statistically analysed with Student's t- test. ns non-significant; * p<0.05

day p.i.	mean % weight on day 0 mean FAP gko (n=7)	mean C57BL/6 (n=7)	p value	p value summary	Significantly different? (P < 0.05)
d7	86.16 ± 2.839	81.17 ± 0.5517	0.1103	ns	no
d8	82.54 ± 3.445	77.09 ± 0.3910	0.1422	ns	no
d9	80.10 ± 3.856	72.43 ± 0.4570	0.072	ns	no
d10	80.67 ± 4.545	70.42 ± 1.098	0.0488	*	yes
d11	82.00 ± 5.225	71.45 ± 2.952	0.1041	ns	no
d12	84.91 ± 4.690	74.99 ± 3.857	0.1282	ns	no
d13	89.89 ± 4.091	81.26 ± 4.995	0.2059	ns	no
d14	92.57 ± 3.108	84.10 ± 4.358	0.1397	ns	no
d15	93.93 ± 2.487	86.06 ± 3.890	0.1141	ns	no
d16	96.88 ± 2.548	90.16 ± 3.285	0.1322	ns	no
